# Supplementary material for: NRF2-dependent Epigenetic Regulation can Promote the Hybrid Epithelial/Mesenchymal Phenotype
Source: Front Cell Dev Biol. 2022 Jan 17;9:828250. doi: 10.3389/fcell.2021.828250 (PMC8803900; doi:10.3389/fcell.2021.828250)
Supplement: Supplementary file 1 [file DataSheet1.PDF]

Supplementary Information

Wen Jia[1,2,5], Mohit Kumar Jolly[3,*], and Herbert Levine[1,4,5,*]

[1]Center for Theoretical Biological Physics, Northeastern University, Boston, MA 02115, USA
[2]Department of Physics and Astronomy, Rice University, Houston, TX 77005, USA
[3]Centre for BioSystems Science and Engineering, Indian Institute of Science, Bangalore 560012, India
[4]Department of Bioengineering, Northeastern University, Boston, MA 02115, US
[5]Department of Physics, Northeastern University, Boston, MA 02115, USA
[*]Correspondence: mkjolly@iisc.ac.in (M.K.J.), h.levine@northeastern.edu (H.L.)

1.Theoretical model for EMT

The EMT network consists of two mutually inhibiting loop: miR-200/ZEB and miR-34/SNAIL. Deterministic equations for miR-200/ZEB circuit with the external signal as SNAIL are given below [1]:

$$\dot{\mu}_{200} = g_{\mu_{200}} H^s(Z) H^s(S) - g_z H^s(Z) H^s(S) H^s(E) P_y(\mu_{200}, 6) - \gamma_\mu \mu_{200}$$
$$\dot{Z} = k_p g_z H^s(Z) H^s(S) H^s(E) P_l(\mu_{200}, 6) - \gamma_z Z$$

and those for miR-34/SNAIL circuit with I as an external signal are:

$$\dot{\mu}_{34} = g_{\mu_{34}} H^s(S) H^s(Z) - g_s H^s(X) H^s(S) H^s(I_{ext}) P_y(\mu_{34}, 2) - \gamma_{\mu_{34}} \mu_{34}$$

$$\dot{S} = k_p g_s H^s(X) H^s(S) H^s(I_{ext}) P_l(\mu_{34}, 2) - \gamma_s S$$

and for the NRF2 session:

$$\dot{K} = k_K H^s(\mu_{200}) - \gamma_K K$$

$$\dot{E} = k_E H^S(Z) - \gamma_E E$$

$$\dot{X} = k_X H^S(K) H^S(E) - \gamma_X X$$

where g is the innate synthesis rate for corresponding microRNA /protein,k_p is the translation rate for ZEB and SNAIL, γ is the corresponding innate degradation rate, and $k_{K,E,X}$ is the single production rate for KEAP1, E-cadherin and NRF2. Here H^S represents the shifted Hill function which is defined as:

$$H^s(B) = \frac{1+\lambda\left(\frac{B}{B_0}\right)^{n_B}}{1+\left(\frac{B}{B_0}\right)^{n_B}}$$

where λ is the fold change regulated by protein B. λ>1 for activation and λ<1 for inhibition.

$P_y(\mu, n)$ describes the decrease in the level of microRNA because of the degradation of the microRNA/mRNA complex. The detailed derivation of these functions in the Supplementary Information of Lu *et al.* [2].

The external signal I that we use here can be written as the stochastic differential equation:

$$\dot{I} = \beta(I_0 - I) + \eta(t)$$

where $\eta(t)$ satisfies the condition that $< \eta(t), n(t') \geq \Gamma\delta(t-t')$. Here I_0 is set at 50 K molecules, β as 0.04 hour^{-1}, and Γ as 1000 (K molecules/hour)^2.
The initial value of I is fixed to lie at the middle of the tristable region {E, E/M, M}.

For the analysis shown in Fig 5, we used the same cell division model described previously [3], where

$$I_{sig}^{daughter} = I_{sig}^{parent} + N(0,1)\eta$$

and

$$B_{0_{sig}}^{daughter} = B_{0_{sig}}^{parent} + N(0,1)\eta.$$

2. Epigenetic feedback regulation term

In the EMT model, we tested epigenetic feedback through three different pathways. The dynamic equation of epigenetic feedback on NRF2's inhibition on SNIAL is:

$$\dot{X}_{ms}^0 = \frac{X_{ms}^0(0) - X_{ms}^0 - \alpha X}{\zeta}$$

The other two epigenetic regulation pathway are modeled by similar method.
where ζ is a timescale factor and chosen to be 100 (hours). α represents the strength of epigenetic feedback. Larger α corresponds to stronger epigenetic feedback. α has an upper bound (usually between 0.01-0.3) because of the restriction that the numbers of all molecules must be positive.

3.Parameters for the EMT model

Table SI 1. List of parameters used in shifted Hill functions

Description	Fold change	Value	# of binding sites	Value	Threshold	Value (K molecules)
**Inhibition on miR-200 by ZEB**	$\lambda_{Z,\mu_{200}}$	0.1	$n_{Z,\mu_{200}}$	3	$Z^0_{\mu_{200}}$	220
**Inhibition on miR-200 by SNAIL**	$\lambda_{S,\mu_{200}}$	0.1	$n_{S,\mu_{200}}$	2	$S^0_{\mu_{200}}$	180
**Self-activation of ZEB**	λ_{Z,m_z}	7.5	n_{Z,m_z}	2	$Z^0_{m_z}$	25
**Activation on ZEB by SNAIL**	λ_{S,m_z}	10.0	n_{S,m_z}	2	$S^0_{m_z}$	180
**Inhibition on miR-34 by SNAIL**	$\lambda_{S,\mu_{34}}$	0.1	$n_{S,\mu_{34}}$	1	$S^0_{\mu_{34}}$	300
**Inhibition on miR-34 by ZEB**	$\lambda_{Z,\mu_{34}}$	0.2	$n_{Z,\mu_{34}}$	2	$Z^0_{\mu_{34}}$	600
**Self-inhibition of SNAIL**	λ_{S,m_s}	0.1	n_{S,m_s}	1	$S^0_{m_s}$	200
**Activation on SNAIL by external signal I**	λ_{I,m_s}	10	n_{I,m_s}	2	$I^0_{m_s}$	50
**Inhibition on SNAIL by NRF2**	λ_{X,m_s}	0.67	n_{X,m_s}	2	$X^0_{m_s}$	1000
**Inhibition on NRF2 by E-cadherin**	$\lambda_{E,X}$	0.33	$n_{E,X}$	2	E^0_X	250
**Inhibition on KEAP1 by miR-200**	λ_{μ_{200},K_s}	0.1	$n_{\mu_{200},K}$	2	${\mu_{200}}^0_K$	5
**Inhibition on NRF2 by KEAP1**	$\lambda_{K,X}$	0.33	$n_{K,X}$	2	$n_{K,X}$	250
**Inhibition on E-cadherin by ZEB**	$\lambda_{Z,E}$	0.1	$n_{Z,E}$	2	$n_{Z,E}$	100
**Inhibition on ZEB by E-cadherin**	λ_{E,m_z}	0.8	n_{E,m_z}	2	n_{E,m_Z}	80

Table SI 2. List of parameters for function Y and L.

n (# of miRNA binding sites)	0	1	2	3	4	5	6
l_i(hour^{-1})	1	0.6	0.3	0.1	0.05	0.05	0.05

γ_{mi}(hour^{-1})	0	0.04	0.2	1	1	1	1
$\gamma_{\mu i}$(hour^{-1})	0	0.005	0.05	0.5	0.5	0.5	0.5
$\boldsymbol{n_{\mu_{200}}}$	6			$n_{\mu_{34}}$			2
$\boldsymbol{\mu_{200}^{0}}$	10K			μ_{34}^{0}			10K

Table SI 3. List of other parameters used in EMT model.

Synthesis rate	Value (10^3molecules/hour)	Degradation rate	Value (hour^{-1})	Production rate	Value (hour^{-1})
$\boldsymbol{g_{\mu_{200}}}$	2.1	$\gamma_{\mu_{200}}$	0.05	k_k	50
$\boldsymbol{g_{\mu_{34}}}$	1.35	γ_z	0.1	k_E	50
$\boldsymbol{g_z}$	0.1	$\gamma_{\mu_{34}}$	0.05	k_X	50
$\boldsymbol{g_s}$	0.1	γ_S	0.125		
		γ_K	0.1		
		γ_X	0.1		
		γ_E	0.1		

4. More results about epigenetic feedback

Epigenetic feedback on the inhibition of NRF2 by KEAP1

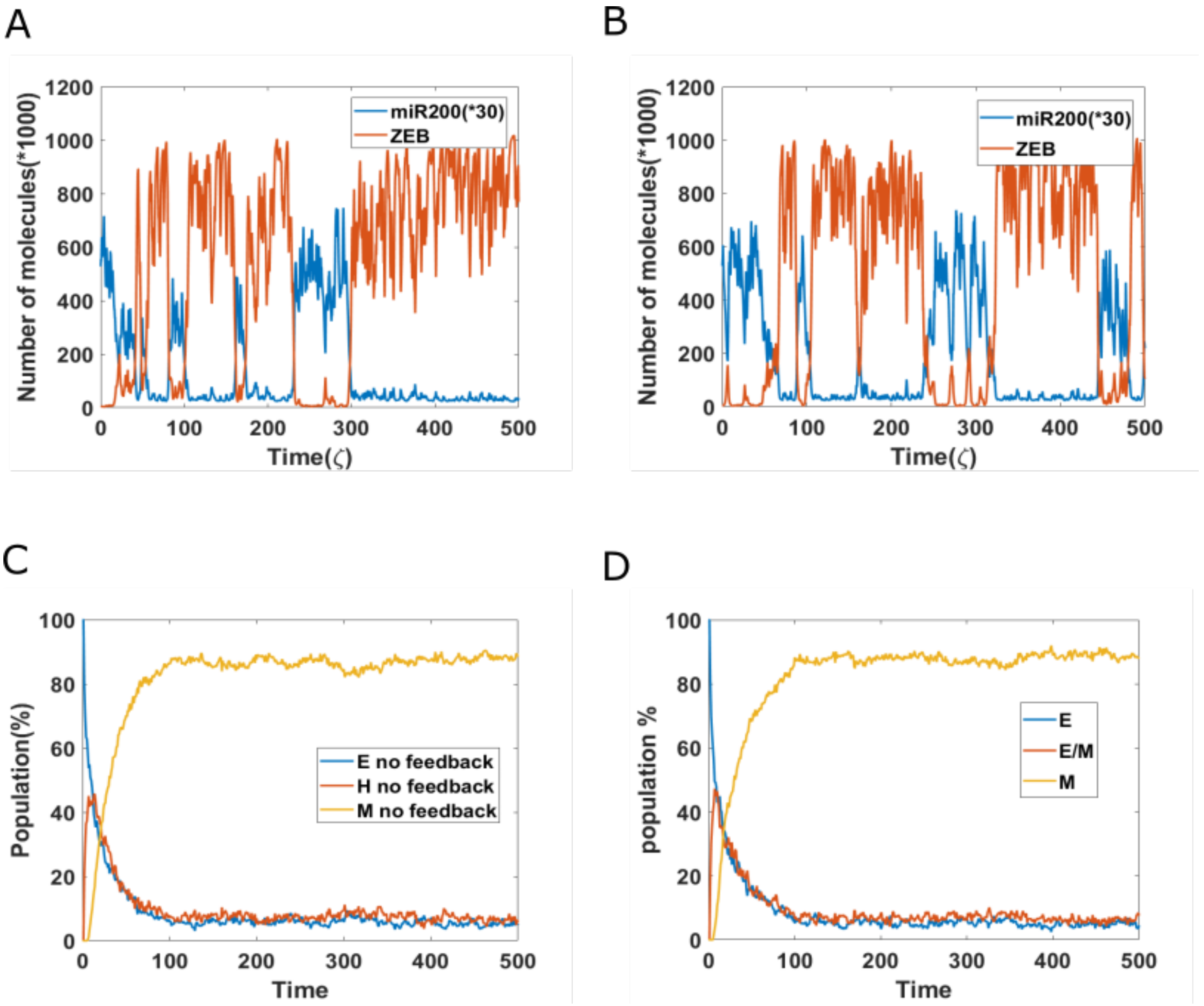

Figure S1: Epigenetic feedback on inhibition of NRF2 by KEAP1. *(A) A sample dynamic plot without epigenetic feedback. (B) A sample dynamic plot with feedback on the inhibition of NRF2 by KEAP1. (C) Simulations showing the population change as a function of time without epigenetic regulation. (D) Same as (C) but now including epigenetic feedback on the inhibitory link from KEAP1 to NRF2.*

Epigenetic feedback on the inhibition of NRF2 by E-cadherin

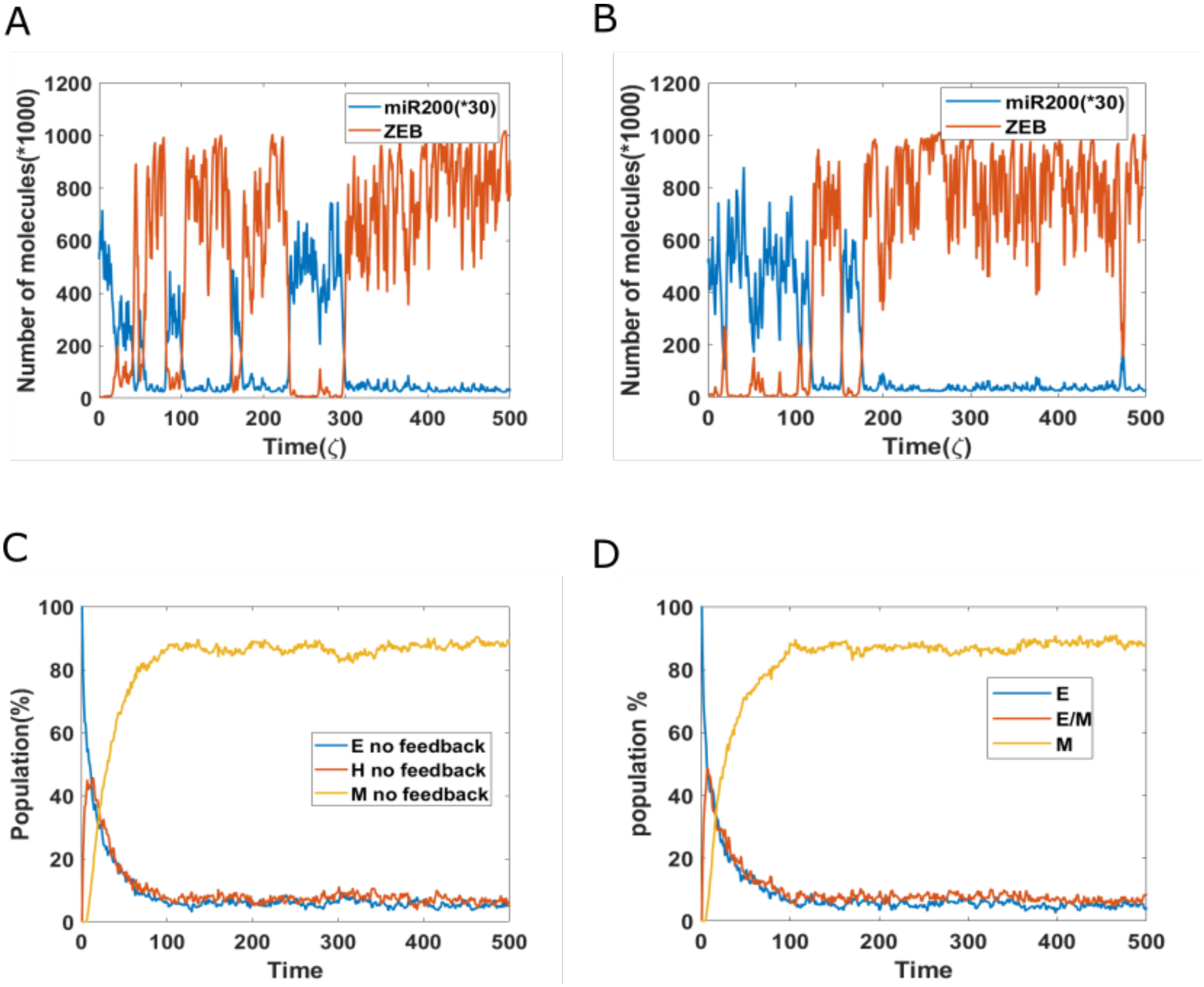

Figure S2: Epigenetic feedback on inhibition of NRF2 by E-cadherin. *(A) A sample dynamic plot without epigenetic feedback. (B) A sample dynamic plot with feedback on the inhibition of NRF2 by E-cadherin. (C) Simulations showing the population change as a function of time without epigenetic regulation. (D) Same as (C) but now including epigenetic feedback on the inhibitory link from E-cadherin to NRF2.*

References

1. Bocci, F.; Tripathi, S. C.; Vilchez, M. S. A.; George, J. T.; Casabar, J.; Wong, P.; Hanash, S.; Levine, H.; Onuchic, J.; Jolly, M. NRF2 activates a partial Epithelial-Mesenchymal Transition and is maximally present in a hybrid Epithelial/Mesenchymal phenotype. *Integr. Biol.* **2019**, *11*, 251–263.
2. Lu, M.; Jolly, M. K.; Levine, H.; Onuchic, J. N.; Ben-Jacob, E. MicroRNA-based regulation of epithelial-hybrid-mesenchymal fate determination. *Proc. Natl. Acad. Sci.* **2013**, *110*, 18144–18149.
3. Tripathi, S.; Chakraborty, P.; Levine, H.; Jolly, M. K. A mechanism for epithelial-mesenchymal heterogeneity in a population of cancer cells. *PLoS Comput Biol* **2020**, *16*, e1007619.
